# Soluble RAGE further stratifies risk of coronary artery and end-stage kidney disease in high-risk individuals with type 1 diabetes and treatment-resistant hypertension

**DOI:** 10.1186/s12933-025-03017-8

**Published:** 2025-12-12

**Authors:** Krishna Adeshara, Raija Lithovius, Stefan Mutter, Valma Harjutsalo, Markku Lehto, Per-Henrik Groop, Niina Sandholm, the FinnDiane Study Group

**Affiliations:** 1 Folkhälsan Research Center, Haartmaninkatu 8, FI-00290 Helsinki, Finland; 2 Department of Nephrology, University of Helsinki and Helsinki University Hospital, Helsinki, Finland; 3 Research Program for Clinical and Molecular Metabolism, Faculty of Medicine, University of Helsinki, Helsinki, Finland; 4 Department of Diabetes, Central Clinical School, Monash University, Melbourne, VIC Australia; 5 Baker Heart and Diabetes Institute, Melbourne, VIC Australia

**Keywords:** Soluble RAGE, Treatment-resistant hypertension, Coronary artery disease, Kidney disease, Type 1 diabetes

## Abstract

**Background:**

Soluble receptor for advanced glycation end-products (sRAGE) modulates RAGE-mediated inflammation and oxidative stress. We investigated if sRAGE stratifies cardiovascular and kidney disease risk in individuals with type 1 diabetes and baseline treatment-resistant hypertension (TRH).

**Methods:**

This study included 1262 adults with type 1 diabetes from the FinnDiane study who were on antihypertensive therapy and whose sRAGE concentration was measured at baseline. Participants were divided into groups: controlled blood pressure (BP) (n = 295), uncontrolled BP (n = 730) or TRH (n = 237). Prospective analyses were performed in those with baseline TRH. Of them, 62 developed coronary artery disease (CAD) and 38 stroke (median follow-up 12 years), while 99 progressed to end-stage kidney disease (ESKD) (median follow-up 9.2 years).

**Results:**

Every 100 units increase in baseline sRAGE was associated with 4% higher odds for TRH, compared to those with uncontrolled BP (*P* = 0.003), and 6% higher odds than those with controlled BP (*P* = 0.0006). Associations attenuated after adjusting for kidney markers. In the competing risk analysis, higher sRAGE was associated with greater risk of CAD (SHR 1.05, *P* = 0.01) in those with TRH. After adjusting for eGFR, the association attenuated (SHR 1.04,* P* = 0.052), but the same trend remained. sRAGE was not associated with stroke. Furthermore, sRAGE was associated with higher risk of ESKD (SHR 1.06, *P* < 0.0001), but no longer after adjusting for eGFR (*P* = 0.4).

**Conclusions:**

Elevated sRAGE is associated with increased odds of TRH in individuals with type 1 diabetes. sRAGE further stratifies high risk of incident CAD and ESKD, even after accounting for clinical variables. Along with eGFR, sRAGE may help to identify individuals at the highest risk of adverse cardiovascular and kidney outcomes.

**Supplementary Information:**

The online version contains supplementary material available at 10.1186/s12933-025-03017-8.

## Research insights

**What is currently known about this topic?**

Soluble RAGE has been linked to various diabetes-related complications. Linking it to hypertension may enhance risk stratification for cardiovascular or kidney disease.

**What is the key research question?**

Is sRAGE linked to TRH in type 1 diabetes, and does it refine cardiovascular and kidney disease risk?

**What is new?**

Higher sRAGE was associated with increased odds of TRH. In TRH, higher sRAGE identified individuals at greater risk for CAD and ESKD.

**How might this study influence clinical practice?**

sRAGE may help improve vascular risk stratification, especially in those with TRH.

## Introduction

Although hypertension is a major contributing risk factor for cardiovascular disease (CVD) and kidney complications in individuals with type 1 diabetes, the majority of individuals treated with antihypertensive drugs fail to reach their recommended blood pressure (BP) targets [1]. A subset of these individuals is designated as having treatment-resistant hypertension (TRH), a condition characterized by failure to achieve the target BP even after using ≥ 3 antihypertensive drugs or BP that requires ≥ 4 antihypertensive drugs to be controlled [34]. Several studies in the general population have demonstrated that TRH is an independent risk factor for all-cause mortality, adverse cardiovascular and kidney outcomes [2, 3]. We have previously shown that TRH is a risk factor for the same outcomes also in type 1 diabetes, although the associations with all-cause mortality and adverse cardiovascular outcomes were attenuated after adjusting for kidney function [4]. Despite being a high-risk phenotype, these individuals with TRH remain poorly characterized and there is a lack of information on how their risk could be further stratified.

Emerging evidence suggests that the receptor for advanced glycation end-products (RAGE) may play a role in the pathophysiology of vascular dysfunction in individuals with diabetes [5]. RAGE is a surface receptor and interacts with multiple proinflammatory ligands, including advanced glycation end products (AGEs). These interactions activate inflammatory processes and an oxidative stress response, key factors involved in the pathogenesis of hypertension [6]. Soluble RAGE (sRAGE) is a truncated, circulating isoform of RAGE that has been shown to act as a decoy receptor for AGEs. However, given the lower endogenous serum concentrations of sRAGE it is unlikely that sRAGE could effectively capture AGEs [5]. Instead, sRAGE more likely reflects increased surface RAGE expression rather than acting as a protective factor.

sRAGE has been linked to various health conditions and outcomes. Increased sRAGE concentrations have been reported to be associated with kidney disease in both type 1 [7] and type 2 diabetes [8]. Elevated sRAGE has also been shown to be associated with eGFR decline in individuals with chronic kidney disease and type 2 diabetes [9], and with all-cause and cardiovascular mortality in type 1 diabetes [10]. Furthermore, an experimental model of diabetic mice deficient in apolipoprotein E (apoE-null) demonstrated that treatment with sRAGE suppressed the development of accelerated diabetic atherosclerosis in a dose-dependent manner [11]. Among people with hypertension in the general population, sRAGE has been described to correlate with central aortic stiffness [12], and inversely with left ventricular hypertrophy [13]. However, very little is known about the association between sRAGE and TRH. A recent study in the general population observed lower sRAGE concentrations in individuals with TRH compared to normotensive controls, but no differences were observed between TRH and mild-hypertension groups [6]. Although these observations suggest a potential implication of RAGE and its isoforms in vascular dysfunction, their specific role with regards to the high-risk TRH phenotype in type 1 diabetes remains unclear. Therefore, in this study we hypothesized that sRAGE, as a modulator of the RAGE-mediated inflammatory response, can further stratify high-risk individuals with type 1 diabetes and TRH with regards to incident coronary artery disease (CAD), stroke, and end-stage kidney disease (ESKD) and thereby identify the individuals at the highest risk.

## Research design and methods

### The study cohort

This study is part of the Finnish Diabetic Nephropathy (FinnDiane) study, an ongoing, nationwide, multicenter study aiming to identify risk factors for diabetic complications in individuals with type 1 diabetes. Type 1 diabetes was defined as age at diabetes onset < 40 years and permanent insulin treatment started within one year after the diagnosis. At baseline, the participants underwent a thorough clinical investigation. BP, body weight, height, waist and hip circumferences were measured, and blood and timed urine samples were collected. BP was measured twice with 2-min intervals in the sitting position after 10 min rest using a mercury sphygmomanometer or an automated standardized BP device. The mean of these two measurements was used in the analyses. Serum sRAGE concentrations were measured by a sandwich ELISA (R&D Biosystems, Minneapolis, MN, USA) according to the manufacturers’ instructions from frozen stored samples. In addition, details of the participants’ clinical characteristics were obtained from medical records and from standardized questionnaires. Each participant completed detailed questionnaires regarding their lifestyle, diet, smoking habits, and family history. Albuminuria was assessed based on two out of three consecutive urine collections. Normal albumin excretion rate (AER) was defined as AER < 20 µg/min or < 30 mg/24 h or ACR (albumin-to-creatinine ratio) < 3.0 mg/mmol; moderate albuminuria as an AER ≥ 20 and < 200 µg/min or ≥ 30 and < 300 mg/24 h or ACR ≥ 3.0 and < 30 mg/mmol; and severe albuminuria as AER ≥ 200 µg/min or ≥ 300 mg/24 h or ACR ≥ 30 mg/mmol. Estimated glomerular filtration rate (eGFR) was calculated from serum creatinine using the 2009 Chronic Kidney Disease Epidemiology Collaboration (CKD-EPI) formula version [14]. The study protocol was approved by the Ethics Committee of the Helsinki and Uusimaa Hospital District, and the study was carried out in accordance with the Declaration of Helsinki. All participants gave their informed written consent.

### Medication information

The FinnDiane data were linked to the Drug Prescription Register (DPR) to obtain information on purchases of antihypertensive drugs within half a year before and after the study visits. Drugs were coded according to the Anatomic Therapeutic Chemical (ATC) classification, based on the 2020 ATC Index Version. Antihypertensive drugs were divided into seven classes: Angiotensin-converting-enzyme (ACE) inhibitors (ATC C09A, C09B), angiotensin II antagonists (C09C, C09D), diuretics (C03, C07BB, C09BA, C09DA), β-blocking agents (C07), calcium channel blockers (C08, C07FB, C09BB, C09DB), imidazoline receptor agonists (C02AC), and alpha-adrenoreceptor antagonists (C02CA). Individuals taking single-pill combinations of antihypertensive drugs were counted as taking separate classes of each drug.

### Classification of hypertension

TRH was defined as systolic BP (SBP) ≥ 130 or diastolic BP (DBP) ≥ 85 mmHg and the use of ≥ 3 antihypertensive drugs, one of which was a diuretic, or SBP < 130 and DBP < 85 mmHg while using ≥ 4 antihypertensive drugs. Controlled BP was defined as ≤ 3 antihypertensive drugs and SBP < 130 and DBP < 85 mmHg, and uncontrolled BP as either SBP ≥ 130 or DBP ≥ 85 mmHg and the use of ≤ 2 antihypertensive drugs or SBP ≥ 130 or DBP ≥ 85 mmHg and the use of 3 drugs without a diuretic in the treatment regimen [15, 34].

### Clinical outcomes

Data on cardiovascular events (i.e., CAD, stroke) were derived from the Finnish Care Register for Health Care until the end of 2015. CAD events included the first acute myocardial infarction (*International Classification of Diseases* [ICD] 8/9 Revisions 410, 412; ICD-10 I21–I23) and coronary bypass graft surgery or coronary angioplasty based on the Nordic Classifications of Surgical Procedures (Supplementary Table S1). Stroke included the first cerebrovascular event (ICD-8/9 430–434, ICD-10 I60–I64). ESKD was defined as dialysis treatment or having received a kidney transplant. Data on progression to ESKD were derived from the individuals’ health care records and multiple national registries until the end of 2015. All deaths, including fatal cardiovascular events, were identified from the Cause of Death Register until 31 December 2015.

### Study participants

**Fig. 1:**
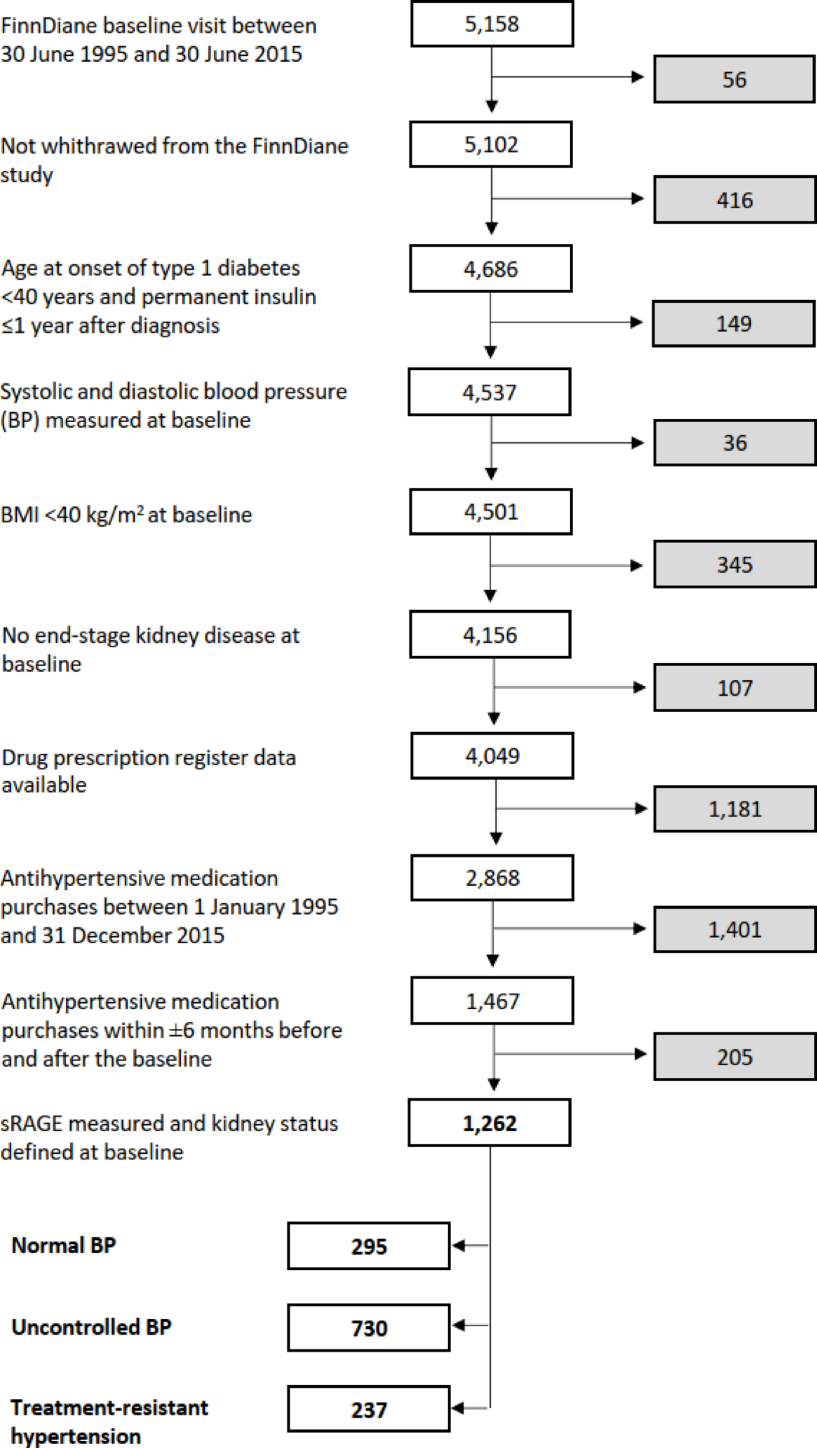
Participant inclusion flowchart

The participant inclusion and exclusion criteria are presented in Fig. 1. We identified 1262 individuals who had completed their FinnDiane baseline visit between 1995 and 2015 and had purchased antihypertensive drugs within half a year before and after the baseline visit. Individuals without sRAGE and systolic and diastolic BP measurements, as well as those with body mass index (BMI) ≥ 40 kg/m^2^ and ESKD at baseline were excluded. Of the cohort, 237 (19%) had TRH, 730 (58%) uncontrolled BP and 295 (23%) controlled BP at baseline. In the prospective analysis, we included only individuals with TRH at baseline and followed them from baseline until their first ever CAD, stroke or ESKD event (each event separately), death, or end of 2015.

### Statistical analysis

Continuous covariates are described as mean ± SD for normally distributed variables and as median with interquartile range (IQR) for non-normally distributed values. Categorical variables are described as percentages. Differences between the groups were tested with t-test or Wilcox signed rank test, for normally and non-normally distributed variables, respectively. Binary variables are expressed as frequency (%) and differences in distributions were tested with Pearson’s chi-squared test or two-tailed Fisher exact test, as appropriate. To investigate the association between sRAGE (as a continuous variable) and TRH at baseline, multivariable logistic regression models were used. To estimate the shapes of the association between sRAGE and the occurrence of TRH, as well as in the prospective phase, the association between sRAGE and outcomes, unadjusted regression models were fitted as a restricted cubic spline. Assumptions of linearity were tested by the Wald test. Cox proportional hazard regression models were used in the prospective analyses. In addition, competing risk analyses by Fine and Gray [16], were applied with death as competing event, and the results are presented as proportional subdistribution hazards ratios (SHR). These models were adjusted for clinical variables, including age, sex, diabetes duration, HbA_1c_, BMI, waist-to-hip ratio or waist-to-height ratio, triglycerides, high-sensitivity C-reactive protein (hs-CRP), current smoking, LDL cholesterol, lipid-lowering drug at baseline, albuminuria status [normal AER, moderate or severe albuminuria], and eGFR, when applicable. To examine whether the effect of sRAGE differed across levels of each risk factors, the interaction term was added in the fully adjusted Cox proportional hazard models. P-values for interaction were obtained from Wald test of the corresponding interaction coefficients. Finally, to compare the effect sizes between different covariates we built Cox proportional and competing risk models by the standardized inputs (per 1SD). To improve interpretability, regression coefficients were expressed per 100 pg/mL increase in sRAGE concentrations (i.e., the original values were divided by 100 prior to model fitting). All statistical analyses were performed with R version 3.5.3 statistical software [35].

## Results

### Clinical characteristics of the study subjects

The mean age was 43.1 ± 11.2 years and the mean duration of diabetes 28.0 ± 10.9 years, and 55% of the participants were men. The baseline characteristics of the participants with respect to the three groups (i.e., TRH, uncontrolled and controlled BP) are shown in Table 1. Individuals with TRH had the worst cardiovascular and kidney disease risk profile of the three groups. They were older and had longer diabetes duration with higher HbA_1c_, BMI and triglycerides, as well as reduced eGFR and more often severe albuminuria than those with uncontrolled or controlled BP. Moreover, those with TRH or uncontrolled BP had higher hs-CRP concentrations, and they were more likely to use lipid-lowering drugs, compared to those with controlled BP. Additionally, the median sRAGE concentration was higher in the individuals with TRH (1316 [IQR 939–1781] pg/ml]), compared to those with uncontrolled BP (1113 [839–1472], *P* < 0.0001) or controlled BP (1145 [859–1495], *P* = 0.002).

**Table 1:** Baseline characteristics of study participants (N = 1262)

Variables	TRH	Uncontrolled BP	Controlled BP	TRH vs. uncontrolled BP	TRH versus controlled BP	Uncontrolled versus controlled BP
N (%)	237 (18.8)	730 (57.8)	295 (23.4)			
Age (years)	46.2 ± 10.3	43.7 ± 11.6	39.4 ± 10.4	0.001	< 0.0001	< 0.0001
Males (%)	65.8	56.6	43.7	0.01	< 0.0001	0.0003
Duration of diabetes (years)	30.2 ± 9.5	28.0 ± 11.4	26.0 ± 10.2	0.003	< 0.0001	0.007
Median diabetes onsetage (years)	13.2 (9.1–22.1)	13.4 (8.6–21.8)	11.7 (7.1–17.7)	0.7	0.002	0.0005
Median sRAGE (pg/ml)	1316 (939–1781)	1113 (839–1472)	1145 (859–1495)	< 0.0001	0.002	0.4
HbA_1c_ (mmol/mol / %)	74 ± 16 / 8.9 ± 1.5	71 ± 16 / 8.6 ± 1.4	71 ± 16 / 8.6 ± 1.5	0.02	0.03	0.9
Systolic BP (mmHg)	152 ± 19	147 ± 15	120 ± 8	< 0.0001	< 0.0001	< 0.0001
Diastolic BP (mmHg)	85 ± 11	84 ± 10	74 ± 7	0.4	< 0.0001	< 0.0001
BMI (kg/m^2^)	26.7 ± 3.5	25.9 ± 3.5	25.2 ± 3.5	0.002	< 0.0001	0.002
Median triglycerides (mmol/L)	1.44 (1.06–2.04)	1.10 (0.80–1.58)	1.04 (0.79–1.50)	< 0.0001	< 0.0001	0.2
LDL cholesterol (mmol/L)	3.32 ± 0.92	3.27 ± 0.85	3.13 ± 0.89	0.4	< 0.0001	0.02
Median hs-CRP (mg/L)	2.47 (1.29–4.94)	2.37 (1.36–4.28)	1.82 (1.17–3.92)	0.4	0.008	0.006
Current or history of smoking (%)	53.0	49.6	53.3	0.4	1.0	0.4
Normal AER (%)	17.7	37.8	32.2	< 0.0001	0.0002	0.1
Moderate albuminuria (%)	16.0	30.3	34.6	< 0.0001	< 0.0001	0.2
Severe albuminuria (%)	66.2	31.9	33.2	< 0.0001	< 0.0001	0.7
eGFR (mL/min/1.73 m^2^)	58 (31–84)	92 (72–105)	91 (71–108)	< 0.0001	< 0.0001	0.6
Lipid-modifying drug purchases at baseline (%)	43.0	26.0	20.0	< 0.0001	< 0.0001	0.05

Data are mean ± standard deviation, median (q1–q3), or %

### sRAGE and TRH at baseline

Higher baseline concentrations of sRAGE increased linearly the odds of TRH at baseline (Table 2, Supplementary Figure S1). In the multivariable logistic regression analysis adjusted for clinical confounders, compared to individuals with uncontrolled BP, each 100 pg/ml increase in sRAGE was associated with 4% higher odds of TRH (*P* = 0.003). When additionally adjusting for eGFR, the association became attenuated (*P* = 0.4). Similarly, compared to those with controlled BP, each 100 pg/ml increase in sRAGE was associated with 6% higher odds of TRH (*P* = 0.0006) after adjustment for clinical confounders, but the association attenuated after additionally adjusting for albuminuria status or eGFR.

**Table 2:** Association between sRAGE (per 100 pg/ml increase) and treatment-resistant hypertension (TRH) (n = 237) compared with uncontrolled BP (n = 730) and controlled BP (n = 295) groups

	TRH versus uncontrolled BP	TRH versus controlled BP
Model	OR (95% CI),* P*	OR (95% CI),* P*
Model 1	1.06 (1.04, 1.08), < 0.0001	1.04 (1.02; 1.07), 0.001
Model 2	1.07 (1.04, 1.09), < 0.0001	1.05 (1.02, 1.08), 0.0004
Model 3	1.07 (1.04, 1.10), < 0.0001	1.06 (1.02, 1.09), 0.0006
Model 4	1.04 (1.01, 1.07), 0.003	1.03 (1.00, 1.06), 0.09
Model 5	1.01 (0.98, 1.04), 0.4	1.01 (0.98, 1.04), 0.6

Model 1 Unadjusted

Model 2 Adjusted for age, sex and duration of diabetes

Model 3 Model 2 + triglycerides, BMI, hs-CRP, HbA_1c_, current smoking, lipid-lowering drug at baseline

Model 4 Model 3 + albuminuria status (i.e., normal AER, moderate or severe albuminuria)

Model 5 Model 4 + eGFR

### sRAGE as future risk indicator in individuals with type 1 diabetes and TRH

For the three prospective analyses, we included 237 individuals with TRH at baseline. In Cox regression analyses, there was a linear relationship between sRAGE and incident CAD (non-linearity *P* = 0.3), incident stroke (non-linearity *P* = 0.6), as well as incident ESKD (non-linearity *P* = 0.1) (Supplementary Figure S2). During a median follow-up of 11.9 (IQR 5.9–15.5) years (2261 person-years) 62 individuals experienced an incident CAD. Similarly, 38 individuals experienced incident stroke event during a median follow-up of 12.0 (IQR 6.7–15.3) years (2348 person-years). Finally, 99 individuals developed ESKD during a median follow-up of 9.2 (IQR 3.5–14.0) years (2060 person-years). As shown in Table 3, each 100 pg/ml increase of the sRAGE concentrations increased the rate of CAD by 5% (*P* = 0.01) after adjusting for age, sex, waist-to-hip ratio, LDL cholesterol, HbA_1c_, current smoking, and eGFR. Effect sizes for all covariates in Model 4 are provided in the supplementary material (Supplementary Table S2). Looking at standardized effect sizes, sRAGE was the fourth strongest risk factor, after smoking, age and the waist-to-hip ratio (Supplementary Table S3). Importantly, the association remained significant even when considering death from other causes than CAD as competing risk (SHR 1.05 [95% CI 1.01, 1.08] *P* = 0.01). Although the association was somewhat attenuated after adjusting for eGFR (SHR 1.04 [95% CI 1.00, 1.07], *P* = 0.052), sRAGE remained a robust predictor of CAD risk.

**Table 3:** sRAGE (as continuous variable per 100 pg/ml increase) and the risk of incident CAD, stroke, and ESKD in individuals with treatment-resistant hypertension (TRH) at baseline

Model	CAD* (n = 210)		Stroke† (n = 217)		ESKD‡ (n = 230)	
Number of events	62		38		99	
Person-years	2261		2348		2060	
Follow-up, years (median [IQR])	11.9 (5.9–15.5)		12.0 (6.7–15.3)		9.2 (3.5–14.0)	
	HR (95% CI), *P*	SHR (95% CI),* P*	HR (95% CI), *P*	SHR (95% CI),* P*	HR (95% CI), *P*	SHR (95% CI),* P*
Model 1	1.03 (1.00, 1.06), 0.03	1.03 (1.00, 1.06), 0.08	1.01 (0.98, 1.05), 0.5	1.01 (0.98, 1.03), 0.7	1.07 (1.05, 1.09), < 0.0001	1.06 (1.03, 1.08), < 0.0001
Model 2	1.05 (1.02, 1.08), 0.003	1.04 (1.00, 1.07), 0.03	1.02 (0.99, 1.05), 0.3	1.01 (0.98, 1.04), 0.5	1.07 (1.04, 1.09), < 0.0001	1.06 (1.03, 1.08), < 0.0001
Model 3	1.06 (1.03, 1.10), 0.0004	1.05 (1.01, 1.08), 0.01	1.01 (0.98, 1.05), 0.4	1.00 (0.98, 1.03),0.8	1.07 (1.04, 1.09), < 0.0001	1.06 (1.03, 1.09), < 0.0001
Model 4	1.05 (1.01, 1.09), 0.01	1.04 (1.00, 1.07), 0.052	0.99 (0.95, 1.03), 0.6	0.98 (0.95, 1.02),0.3	1.04 (1.01, 1.07), 0.02	1.02 (0.98, 1.05), 0.4

Data are presented as hazard ratio (HR) with 95% confidence interval, as well as subhazard ratio (SHR) with 95% confidence interval. *CAD,* coronary artery disease, *ESKD*, end-stage kidney disease

Model: 1 Unadjusted

Model 2: *†Adjusted for age and sex; ‡Adjusted for age, sex and duration of type 1 diabetes

Model 3: *Model 2 + waist-to-hip ratio, LDL cholesterol, HbA_1c_ and current smoking; †Model 2 + BMI and current smoking; ‡ Model 2 + waist-to-height ratio, LDL cholesterol, Triglycerides, HbA_1c_, current smoking

Model 4: *†‡Model 3 + eGFR

Follow-up time > 0.5 years for each event

However, there was an interaction between sRAGE and eGFR in the multivariable Cox model (interaction term: sRAGE × eGFR, *P* = 0.04), indicating that the effect of sRAGE on the hazard was not constant across levels of eGFR. The results were consistent in a competing risk analysis (interaction term: sRAGE × eGFR, *P* = 0.02). Using model-derived estimates from the adjusted Cox model, we found that at low eGFR level (30 mL/min/1.73 m^2^), higher sRAGE was associated with a modestly increased hazard of CAD (HR 1.05 [95% CI 1.01, 1.09], *P* = 0.007), whereas at higher eGFR levels (60 and 90 mL/min/1.73 m^2^), the association was weaker and not statistically significant (*P* = 0.6). Nonethless, sRAGE was not associated with stroke. Furthermore, each 100 pg/ml increase of the sRAGE concentrations increased the rate of ESKD by 4% (*P* = 0.02) after adjustment for clinical confounders. When considering all-cause mortality as a competing risk event, the association remained significant (SHR 1.06 [1.03–1.09], *P* < 0.0001), but was attenuated, when eGFR was added to the model. Regarding of the effect sizes of covariates for ESKD, the lower eGFR emerged as the strongest predictor, while sRAGE was the fifth strongest, after age, male sex, and HbA_1c_. The interaction term between sRAGE and eGFR was non-significant for stroke (sRAGE × eGFR, *P* = 0.2) and for ESKD (sRAGE × eGFR, *P* = 0.97), indicating that the effect of sRAGE on the hazard was constant across levels of eGFR.

## Discussion

In the current study we have in cross-sectional analyses demonstrated that the odds of TRH increased with increasing sRAGE concentrations, compared to those with uncontrolled and controlled BP. Notably, these associations persisted even after adjusting for well-known clinical risk factors, and only eGFR attenuated the association. Importantly, in the prospective analyses in those high-risk individuals with baseline TRH, sRAGE was associated with incident CAD, and the association remained when taking the competing risk of death from other causes than CAD into account. Of note, the association was modified by eGFR and only remained significant in those individuals with a lower eGFR. Therefore, our findings suggest that sRAGE may be an additional biomarker for CAD risk in those with impaired kidney function only.

Similarly, elevated sRAGE was associated with higher risk of ESKD, but not beyond reduced kidney function. In contrast, our study did not find any significant associations between sRAGE and stroke. To the best of our knowledge, this is the first study that shows the potential of sRAGE to further stratify risk of adverse outcomes in an already high-risk population with type 1 diabetes and TRH.

Studies have shown conflicting results regarding the circulating sRAGE concentrations across different health conditions and outcomes. We and others have shown elevated sRAGE concentrations in type 1 and type 2 diabetes along with complications such as CAD and diabetic nephropathy [7, 17, 18], whereas in the general population, reduced sRAGE concentrations have been associated with the metabolic syndrome [19], essential hypertension and left ventricular hypertrophy [13], arterial stiffness [20], and impaired glucose metabolism [21]. Additionally, a study by Gryszczyńska et al. reported lower sRAGE in TRH vs. normotensive controls, with no differences between TRH and mild-hypertension groups in the general population [6]. Conversely, in the present study we found a positive association between sRAGE and TRH in individuals with type 1 diabetes. These findings may suggest that sRAGE concentrations are not uniformly altered in all hypertensive conditions. Furthermore, in relation to CAD, lower sRAGE was correlated with higher pulse pressure in never-treated patients with essential hypertension [22], while higher sRAGE was associated with central aortic stiffness in hypertensive individuals with diabetes [12]. Similarly in the present study elevated sRAGE was associated with increased risk of CAD in those with TRH. These findings align with the notion that differences in disease etiology might explain these associations. sRAGE is a marker of endothelial RAGE expression and systemic inflammation, potentially linking it to CAD through inflammation-specific mechanisms that elevate the CAD risk. These findings thus suggest that sRAGE could be a biomarker of RAGE-mediated inflammation and could potentially improve the risk stratification of vascular complications in diabetes.

Lower sRAGE concentrations were associated with cerebral microbleeds [23], and severe leukoaraiosis in individuals with acute stroke [24], while higher sRAGE concentrations correlated with an unfavorable functional outcome in individuals with ischemic stroke and dementia [25]. Additionally, higher sRAGE concentrations were found in cardioembolic strokes compared to other ischemic stroke subtypes [26]. This suggests that the relationship between sRAGE and stroke may be subtype dependent. In our study, we did not observe any significant association between sRAGE and stroke in individuals with TRH. It is important to note that from our register-based data, we cannot distinguish between different stroke subtypes, which may explain the lack of association. Furthermore, the mechanisms how sRAGE influences cardiovascular events may not overlap with those leading to cerebrovascular events. A study by Tang et al. [25] found that sRAGE concentrations exhibit a dynamic pattern following the stroke onset; initially the sRAGE concentrations were high but decreased going from the acute to the subacute phase. Thus, higher sRAGE concentrations at the onset (i.e., within 48 h) are associated with poor functional outcomes. Similarly, a study by Chu et al. [27] demonstrated that sRAGE concentrations were higher in individuals with aneurysmal subarachnoid hemorrhage than in controls, with decreasing concentrations over time, where early concentrations were linked with the disease severity scores and delayed cerebral ischemia. This dynamic behavior of sRAGE could explain the lack of association in this study. Therefore, further studies are needed to investigate the associations between sRAGE and different subtypes of stroke.

In our cohort, we have previously shown that two-thirds of the individuals with TRH had pre-existing impaired kidney function, as evidenced by reduced eGFR and increased albuminuria [4]. Population studies show high rates of hypertension among CKD patients [3, 28], while a clinical study reported a 23% prevalence of apparent TRH in a white CKD population [29]. Notably, individuals with apparent TRH had 2.3-fold faster decline in kidney function compared to non-resistant arterial hypertension [28]. There is a complex relationship between a declining kidney function and circulating sRAGE; as eGFR decreases, the sRAGE filtration diminishes while the production may increase due to inflammation and tissue damage [8, 12, 30]. This inverse association between sRAGE and eGFR has been observed in various populations, including advanced CKD [31], older community-dwelling women [32], and non-diabetic individuals with CKD [30]. Our group has also found a negative correlation between serum sRAGE and eGFR in individuals with diabetic nephropathy and type 1 diabetes [7]. The present study revealed that higher sRAGE concentrations were associated with increased risk of ESKD in high-risk individuals with TRH. However, the strength of this association was attenuated when eGFR was included in the model, suggesting that the relationship is partly mediated through kidney function decline. This is further underscored by the fact that in our cohort 66% of the individuals with TRH had severe albuminuria, highlighting the significant impact of kidney dysfunction. This finding suggests that sRAGE could serve as an additional biomarker for risk stratification in individuals with declining kidney function, particularly in those with TRH.

The main strengths of our study are the well-characterized participants with type 1 diabetes, a prospective study design, and access to high-quality and complete national registry data. There are, however, limitations regarding the assessment of TRH. First, BP measurements were based on two office-based measurements at a single visit, which may not necessarily reflect out of office BP values. While data on antihypertensive medications were retrieved from the Finnish Drug Prescription Register whose coverage and accuracy is very high [33], doses and frequencies are not recorded. Therefore, we were not able to confirm whether the antihypertensive drugs were administered at the maximum tolerated doses. Consequently, our definition of TRH reflects apparent TRH, and may therefore overestimate the true prevalence of TRH. Nonetheless, the complete coverage of the drug register allows for careful characterization of the types of medications purchased from pharmacies. Importantly, the study setting does not allow any conclusions about causality.

In conclusion, higher sRAGE was associated with increased odds of TRH at baseline compared to those with uncontrolled and controlled BP. sRAGE also showed its potential to further stratify individuals with type 1 diabetes and TRH at higher risk of CAD and ESKD. The association with CAD was independent of clinical factors and only marginally attenuated after adjustment for eGFR, suggesting that sRAGE may provide additional value in identifying individuals at high risk of CAD especially among those with impaired kidney function.

## Supplementary Information

Below is the link to the electronic supplementary material.

Supplementary Material 1.

## Data Availability

Individual-level data for the study participants are not publicly available because of the restrictions due to the study consent provided by the participant at the time of data collection.

## Abbreviations

ACE: Angiotensin-converting-enzyme
ACR: Albumin-to-creatinine ratio
AER: Albumin excretion rate
AGEs: Advanced glycation end products
BMI: Body mass index
BP: Blood pressure
CAD: Coronary artery disease
CVD: Cardiovascular disease
ESKD: End-stage kidney disease
eGFR: Estimated glomerular filtration rate
sRAGE: Soluble receptor for advanced glycation end-products
HbA_1c_: Glycated hemoglobin
hs-CRP: high-sensitivity C-reactive protein
IQR: Interquartile range
SHR: Subdistribution hazards ratios
TRH: Treatment-resistant hypertension

## Declarations

### Clinical trial number

Not applicable. This study has not been registered as a clinical trial.
