# Supplementary material for: Soluble RAGE further stratifies risk of coronary artery and end-stage kidney disease in high-risk individuals with type 1 diabetes and treatment-resistant hypertension
Source: Cardiovasc Diabetol. 2025 Dec 12;25:13. doi: 10.1186/s12933-025-03017-8 (PMC12805710; doi:10.1186/s12933-025-03017-8)
Supplement: Supplementary file 1 — Supplementary Material 1. [file 12933_2025_3017_MOESM1_ESM.docx]

**Supplementary Material**

Krishna Adeshara, Raija Lithovius, Stefan Mutter, Valma Harjutsalo, Markku Lehto, Per-Henrik Groop, Niina Sandholm, on behalf of the FinnDiane Study Group. **Soluble RAGE further stratifies risk of coronary artery and end-stage kidney disease in high-risk individuals with type 1 diabetes and treatment-resistant hypertension**

**Contents**

[**Supplementary Table S1.** ICD and procedure codes used for ascertaining coronary artery disease (CAD) and stroke 2](#_Toc210131789)

[**Supplementary Table S2**. Multivariable Cox proportional and Competing risk models of soluble RAGE and other clinical covariates for risk of coronary artery disease (CAD), stroke, and end-stage kidney disease (ESKD) in individuals with treatment-resistant hypertension at baseline 3](#_Toc210131790)

[**Supplementary Table S3**. Standardized associations (per 1SD) of soluble RAGE and other clinical covariates with incidence coronary artery disease (CAD), stroke and end-stage kidney disease (ESKD) in individuals with treatment-resistant hypertension at baseline: Multivariable Cox proportional and Competing risk models 4](#_Toc210131791)

[**Supplementary Table S4.** Physicians and nurses at each of the FinnDiane centers participating in patient recruitment and characterization 5](#_Toc210131792)

[**Fig. S1.** Association between soluble RAGE and treatment-resistant hypertension in individuals with type 1 diabetes, when controlled BP (**A**) or uncontrolled BP (**B**) was a reference group. Unadjusted logistic regression models were fitted as restricted cubic splines. 7](#_Toc210131793)

[**Fig. S2.** Association between soluble RAGE and incident coronary artery disease (**A**), stroke (**B**) and end-stage kidney disease (C) in individuals with type 1 diabetes and treatment-resistant hypertension. Unadjusted Cox regression models were fitted as a restricted cubic splines. 8](#_Toc210131794)

# **Supplementary Table S1.** ICD and procedure codes used for ascertaining coronary artery disease (CAD) and stroke

| **Code type** |  | **Register** | **Codes** | **Explanation** |
| --- | --- | --- | --- | --- |
| ICD-10 |  | Finnish Causes of Death Register and Finnish Care Register for Health Care (Hospital Discharge Register until 1993) | I21, I22, I23 | myocardial infraction |
|  |  |  | I60, I61, I62, I63, I64 | stroke |
| ICD-9 |  |  | 410, 412 | myocardial infraction |
|  |  |  | 430, 431, 432, 433, 434 | stroke |
| Procedure code |  | Care Register for Health Care (Hospital Discharge Register until 1993) | FNA01, FNA02, FNA03, FNA04, FNA05, FNA10, FNA20, FNA96  FNB01, FNB02, FNB20, FNB96  FNC10, FNC20, FNC30, FNC40, FNC50, FNC60, FNC96  FND10, FND20, FND96  FNE01, FNE02, FNE03, FNE10, FNE11, FNE20, FNE21, FNE96 | coronary bypass surgery (GABG) |
|  |  | Care Register for Health Care (Hospital Discharge Register until 1993) | FN1AT, FN1BT, FN1YT  TFN40,TFN50 | coronary balloon angioplasty (PTCA/PCI) |
|  |  | Care Register for Health Care (Hospital Discharge Register until 1993) | 5311, 5312, 5313, 5314, 5315, 5329 | coronary operations (coronary bypass surgery or balloon angioplasty) before 1996 |

# **Supplementary Table S2**. Multivariable Cox proportional and Competing risk models of soluble RAGE* and other clinical covariates for risk of coronary artery disease (CAD), stroke, and end-stage kidney disease (ESKD) in individuals with treatment-resistant hypertension at baseline

| **CAD** | | |
| --- | --- | --- |
| ***Variables*** | ***HR (95% CI), P*** | ***SHR (95% CI), P*** |
| sRAGE, pg/ml | 1.05 (1.01, 1.09), 0.01 | 1.04 (1.00, 1.07), 0.05 |
| Age, years | 1.06 (1.02, 1.09), 0.0006 | 1.04 (1.01, 1.07), 0.005 |
| Female sex | 1.59 (0.77, 3.30) ,0.2 | 1.49 (0.72, 3.06), 0.3 |
| Waist-to-hip ratio | 1.67 (1.14, 2.43), 0.008 | 1.57 (1.10, 2.25), 0.01 |
| LDL cholesterol, mmol/l | 1.15 (0.82, 1.60), 0.4 | 1.21 (0.85, 1.73), 0.3 |
| HbA_1c,_ mmol/mol | 0.99 (0.98, 1.01), 0.4 | 0.99 (0.97, 1.01), 0.4 |
| Current smoking | 2.10 (1.15, 3.82), 0.02 | 1.97 (1.05, 3.72), 0.04 |
| eGFR, ml/min/1.73 m^2^ | 0.99 (0.98, 1.00), 0.02 | 0.99 (0.98, 1.00), 0.09 |
| **CAD (**Models inteaction term: sRAGE × eGFR**)** | | |
| ***Variables*** | ***HR (95% CI), P*** | ***SHR (95% CI), P*** |
| sRAGE × eGFR | 1.00 (1.00. 1.00), 0.04 | 1.00 (1.00, 1.00), 0.02 |
| sRAGE, pg/ml | 1.09 (1.04, 1.15), 0.0006 | 1.09 (1.04, 1.15), 0.0009 |
| Age, years | 1.05 (1.02, 1.09), 0.001 | 1.04 (1.01, 1.07), 0.006 |
| Female sex | 1.72 (0.82, 3.58), 0.1 | 1.65 (0.78, 3.49), 0.2 |
| Waist-to-hip ratio | 1.56 (1.07, 2.28), 0.02 | 1.47 (1.02, 2.12), 0.04 |
| LDL cholesterol, mmol/l | 1.15 (0.82, 1.62), 0.4 | 1.23 (0.85, 1.77), 0.3 |
| HbA_1c,_ mmol/mol | 0.99 (0.98, 1.01), 0.5 | 0.99 (0.97, 1.01), 0.4 |
| Current smoking | 2.11 (1.15, 3.85), 0.02 | 2.00 (1.06, 3.76), 0.03 |
| eGFR, ml/min/1.73 m^2^ | 1.00 (1.00, 1.02), 0.7 | 1.01 (0.66, 1.03), 0.3 |
| **Stroke** | | |
| ***Variables*** | ***HR (95% CI), P*** | ***SHR (95% CI), P*** |
| sRAGE, pg/ml | 0.99 (0.95, 1.03), 0.6 | 0.98 (0.95, 1.02), 0.3 |
| Age, years | 1.04 (1.01, 1.08), 0.02 | 1.03 (0.99, 1.07), 0.09 |
| Female sex | 0.69 (0.33, 1.42), 0.3 | 0.74 (0.36, 1.51), 0.4 |
| BMI, kg/m^2^ | 0.90 (0.81, 0.99), 0.04 | 0.90 (0.81, 1.00), 0.04 |
| Current smoking | 1.37 (0.60, 3.11), 0.5 | 1.08 (0.44, 2.65), 0.9 |
| eGFR, ml/min/1.73 m^2^ | 0.98 (0.96, 0.99), 0.0004 | 0.98 (0.97, 1.00), 0.006 |
| **ESKD** | | |
| ***Variables*** | ***OR (95% CI), P*** | ***SHR (95% CI), P*** |
| sRAGE, pg/ml | 1.04 (1.01, 1.07), 0.02 | 1.02 (0.98, 1.05), 0.4 |
| Age, years | 0.95 (0.92, 0.98), 0.0006 | 0.96 (0.94, 0.99), 0.002 |
| Female sex | 0.60 (0.37, 0.98), 0.04 | 0.64 (0.42, 0.99), 0.05 |
| Duration of diabetes, years | 1.03 (0.99, 1.06), 0.2 | 1.00 (0.96, 1.03), 0.8 |
| Waist-to-hip ratio | 1.11 (0.81, 1.53), 0.5 | 0.95 (0.70, 1.27), 0.7 |
| LDL cholesterol, mmol/l | 1.03 (0.78, 1.36), 0.9 | 0.96 (0.71, 1.32), 0.8 |
| Triglycerides, mmol/l | 0.98 (0.78, 1.22), 0.9 | 0.92 (0.74, 1.15), 0.5 |
| HbA_1c,_ mmol/mol | 1.03 (1.01, 1.05), 0.001 | 1.02 (1.00, 1.04), 0.04 |
| Current smoking | 1.53 (0.88, 2.66), 0.1 | 1.46 (0.83, 2.54), 0.2 |
| eGFR, ml/min/1.73 m^2^ | 0.92 (0.90, 0.93), <0.0001 | 0.92 (0.91, 0.94), <0.0001 |

*Each 100pg/ml increase; Data are presented as hazard ratio (HR) with 95% confidence interval, as well as subhazard ratio (SHR) with 95% confidence interval

# **Supplementary Table S3**. Standardized associations (per 1SD) of soluble RAGE and other clinical covariates with incidence coronary artery disease (CAD), stroke and end-stage kidney disease (ESKD) in individuals with treatment-resistant hypertension at baseline: Multivariable Cox proportional and Competing risk models

| **CAD** | | |
| --- | --- | --- |
| ***Variables*** | ***HR (95% CI), P*** | ***SHR (95% CI), P*** |
| Current smoking | 2.10 (1.15, 3.82), 0.02 | 1.97 (1.05, 3.72), 0.04 |
| Age, years | 1.67 (1.25, 2.25), 0.0006 | 1.48 (1.13, 1.95), 0.005 |
| Waist-to-hip ratio | 1.62 (1.13, 2.31), 0.008 | 1.53 (1.09, 2.15), 0.01 |
| sRAGE, pg/ml | 1.42 (1.07, 1.89), 0.01 | 1.30 (1.00, 1.70), 0.05 |
| eGFR, ml/min/1.73 m^2^ | 0.66 (0.47, 0.93), 0.02 | 0.73 (0.52, 1.04), 0.09 |
| Male sex | 0.63 (0.30, 1.30) ,0.2 | 0.67 (0.33, 1.38), 0.3 |
| LDL cholesterol, mmol/l | 1.13 (0.83, 1.54), 0.4 | 1.19 (0.86, 1.65), 0.3 |
| HbA_1c,_ mmol/mol | 0.89 (0.67, 1.19), 0.4 | 0.88 (0.66, 1.17), 0.4 |
| **Stroke** | | |
| ***Variables*** | ***HR (95% CI), P*** | ***SHR (95% CI), P*** |
| eGFR, ml/min/1.73 m^2^ | 0.48 (0.32, 0.72), 0.0004 | 0.54 (0.35, 0.84), 0.006 |
| Age, years | 1.50 (1.06, 2.12), 0.02 | 1.35 (0.96, 1.90), 0.09 |
| BMI, kg/m^2^ | 0.68 (0.48, 0.98), 0.04 | 0.69 (0.48, 0.99), 0.04 |
| Male sex | 1.45 (0.71, 2.99), 0.3 | 1.36 (0.66, 2.77), 0.4 |
| Current smoking | 1.37 (0.60, 3.11), 0.5 | 1.08 (0.44, 2.65), 0.9 |
| sRAGE, pg/ml | 0.92 (0.63, 1.32), 0.6 | 0.86 (0.63, 1.18), 0.3 |
| **ESKD** | | |
| ***Variables*** | ***OR (95% CI), P*** | ***SHR (95% CI), P*** |
| eGFR, ml/min/1.73 m^2^ | 0.06 (0.03, 0.11), <0.0001 | 0.08 (0.05, 0.15), <0.0001 |
| Age, years | 0.59 (0.44, 0.80), 0.0006 | 0.68 (0.53, 0.87), 0.002 |
| Male sex | 1.67 (1.02, 2.72), 0.04 | 1.55 (1.01, 2.39), 0.05 |
| HbA_1c,_ mmol/mol | 1.53 (1.19, 1.98), 0.001 | 1.33 (1.02, 1.74), 0.04 |
| sRAGE, pg/ml | 1.31 (1.04, 1.64), 0.02 | 1.12 (0.85, 1.48), 0.4 |
| Current smoking | 1.53 (0.88, 2.66), 0.1 | 1.46 (0.83, 2.54), 0.2 |
| Duration of diabetes, years | 1.28 (0.91, 1.79), 0.2 | 0.97 (0.68, 1.37), 0.8 |
| Waist-to-hip ratio | 1.07 (0.87, 1.31), 0.5 | 0.97 (0.80, 1.17), 0.7 |
| Triglycerides, mmol/l | 0.98 (0.76, 1.25), 0.9 | 0.91 (0.71, 1.17), 0.5 |
| LDL cholesterol, mmol/l | 1.02 (0.80, 1.31), 0.9 | 0.97 (0.74, 1.28), 0.8 |

Data are presented as hazard ratio (HR) with 95% confidence interval, as well as subhazard ratio (SHR) with 95% confidence interval.Variables are standardized and presented in descending order of relative importance based on effect sizes.

# **Supplementary Table S4.** Physicians and nurses at each of the FinnDiane centers participating in patient recruitment and characterization

**The Finnish Diabetic Nephropathy Study Center Physicians and nurses**

Anjalankoski Health Center S.Koivula, T.Uggeldahl

Central Finland Central Hospital, Jyväskylä T.Forslund, A.Halonen, A.Koistinen, P.Koskiaho, M.Laukkanen, J.Saltevo, M.Tiihonen

Central Hospital of Åland Islands, Mariehamn M.Forsen, H.Granlund, A.-C.Jonsson, B.Nyroos

Central Hospital of Kanta-Häme, Hämeenlinna P.Kinnunen, A.Orvola, T.Salonen, A.Vähänen

Central Hospital of Kymenlaakso, Kotka R.Paldanius, M.Riihelä, L.Ryysy

Central Hospital of Länsi-Pohja, Kemi H.Laukkanen, P.Nyländen, A.Sademies

Central Ostrobothnian Hospital District, Kokkola S.Anderson, B.Asplund, U.Byskata, P.Liedes, M.Kuusela, T.Virkkala

City of Espoo Health Center:

Espoonlahti A.Nikkola, E.Ritola

Tapiola M.Niska, H.Saarinen

Samaria E.Oukko-Ruponen, T.Virtanen

Viherlaakso A.Lyytinen

City of Helsinki Health Center:

Puistola H.Kari, T.Simonen

Suutarila A.Kaprio, J.Kärkkäinen, B.Rantaeskola

Töölö P.Kääriäinen, J.Haaga, A-L.Pietiläinen

City of Hyvinkää Health Center S.Klemetti, T.Nyandoto, E.Rontu, S.Satuli-Autere

City of Vantaa Health Center:

Korso R.Toivonen, H.Virtanen

Länsimäki R.Ahonen, M.Ivaska-Suomela, A.Jauhiainen

Martinlaakso M.Laine, T.Pellonpää, R.Puranen

Myyrmäki A.Airas, J.Laakso, K.Rautavaara

Rekola M.Erola, E.Jatkola

Tikkurila R.Lönnblad, A.Malm, J.Mäkelä, E.Rautamo

Heinola Health Center P.Hentunen, J.Lagerstam

Helsinki University Central Hospital, Department of M.Feodoroff, D.Gordin, O.Heikkilä, K.Hietala, J.Fagerudd, M.Korolainen, Medicine, Division of Nephrology L.Kyllönen, J.Kytö, S.Lindh, K.Pettersson-Fernholm, M.Rosengård-Bärlund, A.Sandelin, L.Thorn, J.Tuomikangas, T.Vesisenaho, J.Wadén

Herttoniemi Hospital, Helsinki V.Sipilä

Hospital of Lounais-Häme, Forssa T.Kalliomäki, J.Koskelainen, R.Nikkanen, N.Savolainen, H.Sulonen, E.Valtonen

Hyvinkää Hospital L. Norvio, A.Hämäläinen

Iisalmi Hospital E.Toivanen

Jokilaakso Hospital, Jämsä A.Parta, I.Pirttiniemi

Jorvi Hospital, Helsinki University Central Hospital S.Aranko, S.Ervasti, R.Kauppinen-Mäkelin, A.Kuusisto, T.Leppälä, K.Nikkilä, L.Pekkonen

Jyväskylä Health Center, Kyllö K.Nuorva, M.Tiihonen

Kainuu Central Hospital, Kajaani S.Jokelainen, K.Kananen, M.Karjalainen, P.Kemppainen, A-M.Mankinen, A.Reponen, M.Sankari

Kerava Health Center H.Stuckey, P.Suominen

Kirkkonummi Health Center A.Lappalainen, M.Liimatainen, J.Santaholma

Kivelä Hospital, Helsinki A.Aimolahti, E.Huovinen

Koskela Hospital, Helsinki V.Ilkka, M.Lehtimäki

Kotka Health Center E.Pälikkö-Kontinen, A.Vanhanen

Kouvola Health Center E.Koskinen, T.Siitonen

Kuopio University Hospital E.Huttunen, R.Ikäheimo, P.Karhapää, P.Kekäläinen, M.Laakso, T.Lakka, E.Lapmpainen, L.Moilanen, S. Tanskanen, L.Niskanen, U.Tuovinen,

I.Vauhkonen, E.Voutilainen, U.Tuovinen, I.Vauhkonen,

Kuusamo Health Center T.Kääriäinen, E.Isopoussu

Kuusankoski Hospital E.Kilkki, I.Koskinen, L.Riihelä

Laakso Hospital, Helsinki T.Meriläinen, P.Poukka, R.Savolainen, N.Uhlenius

Lahti City Hospital A.Mäkelä, M.Tanner

Lapland Central Hospital, Rovaniemi L.Hyvärinen, K.Lampela, S.Pöykkö, T.Rompasaari, S.Severinkangas, T.Tulokas

Lappeenranta Health Center P. Erola, L.Härkönen, P.Linkola, T.Pekkanen, I.Pulli, E.Repo

Lohja Hospital T.Granlund, K.Hietanen, M.Porrassalmi, M.Saari, T.Salonen, M.Tiikkainen,

Länsi-Uusimaa Hospital, Tammisaari I.-M.Jousmaa, J.Rinne

Loimaa Health Center A.Mäkelä, P.Eloranta

Malmi Hospital, Helsinki H.Lanki, S.Moilanen, M.Tilly-Kiesi

Mikkeli Central Hospital A.Gynther, R.Manninen, P.Nironen, M.Salminen, T.Vänttinen

Mänttä Regional Hospital I.Pirttiniemi, A-M.Hänninen

North Karelian Hospital, Joensuu U-M.Henttula, P.Kekäläinen, M.Pietarinen, A.Rissanen, M.Voutilainen

Nurmijärvi Health Center A.Burgos, K.Urtamo

Oulaskangas Hospital, Oulainen E.Jokelainen, P-L.Jylkkä, E.Kaarlela, J.Vuolaspuro

Oulu Health Center L.Hiltunen, R.Häkkinen, S.Keinänen-Kiukaanniemi

Oulu University Hospital R.Ikäheimo

Päijät-Häme Central Hospital H.Haapamäki, A.Helanterä, S.Hämäläinen, V.Ilvesmäki, H.Miettinen

Palokka Health Center P.Sopanen, L.Welling

Pieksämäki Hospital V.Sevtsenko, M.Tamminen

Pietarsaari Hospital M-L.Holmbäck, B.Isomaa, L.Sarelin

Pori City Hospital P.Ahonen, P.Merisalo, E.Muurinen, K.Sävelä

Porvoo Hospital M.Kallio, B.Rask, S.Rämö

Raahe Hospital A.Holma, M.Honkala, A.Tuomivaara, R.Vainionpää

Rauma Hospital K.Laine, K.Saarinen, T.Salminen

Riihimäki Hospital P.Aalto, E.Immonen, L.Juurinen

Salo Hospital A.Alanko, J.Lapinleimu, P.Rautio, M.Virtanen

Satakunta Central Hospital, Pori M.Asola, M.Juhola, P.Kunelius, M.-L.Lahdenmäki, P.Pääkkönen, M.Rautavirta

Savonlinna Central Hospital T.Pulli, P.Sallinen, M.Taskinen, E.Tolvanen, T.Tuominen, H.Valtonen,

A.Vartia, S-L. Viitanen

Seinäjoki Central Hospital O.Antila, E.Korpi-Hyövälti, T.Latvala, E.Leijala, T.Leikkari, M.Punkari, ,

N.Rantamäki, H.Vähävuori

South Karelia Central Hospital, Lappeenranta T.Ensala, E.Hussi, R.Härkönen, U.Nyholm, J.Toivanen

Tampere Health Center A.Vaden, P.Alarotu, E.Kujansuu, H.Kirkkopelto-Jokinen, M.Helin,

S.Gummerus, L.Calonius, T.Niskanen, T.Kaitala, T.Vatanen

Tampere University Hospital P. Hannula, I.Ala-Houhala, R.Kannisto, T.Kuningas, P.Lampinen, M.Määttä,

H.Oksala, T.Oksanen, A.Putila, H.Saha, K.Salonen, H.Tauriainen, S.Tulokas

Tiirismaa Health Center, Hollola T.Kivelä, L.Petlin, L.Savolainen

Turku Health Center A.Artukka, I.Hämäläinen, L.Lehtinen, E.Pyysalo, H.Virtamo, M.Viinikkala,

M.Vähätalo

Turku University Central Hospital K.Breitholz, R.Eskola, K.Metsärinne, U.Pietilä, P.Saarinen, R.Tuominen,

S.Äyräpää

Vaajakoski Health Center K.Mäkinen, P.Sopanen

Valkeakoski Regional Hospital S.Ojanen, E.Valtonen, H.Ylönen, M.Rautiainen,T.Immonen

Vammala Regional Hospital I.Isomäki, R.Kroneld, L.Mustaniemi, M.Tapiolinna-Mäkelä

Vaasa Central Hospital S.Bergkulla, U.Hautamäki, V-A.Myllyniemi, I.Rusk

# **Fig. S1.** Association between soluble RAGE and treatment-resistant hypertension in individuals with type 1 diabetes, when controlled BP (**A**) or uncontrolled BP (**B**) was a reference group. Unadjusted logistic regression models were fitted as restricted cubic splines.


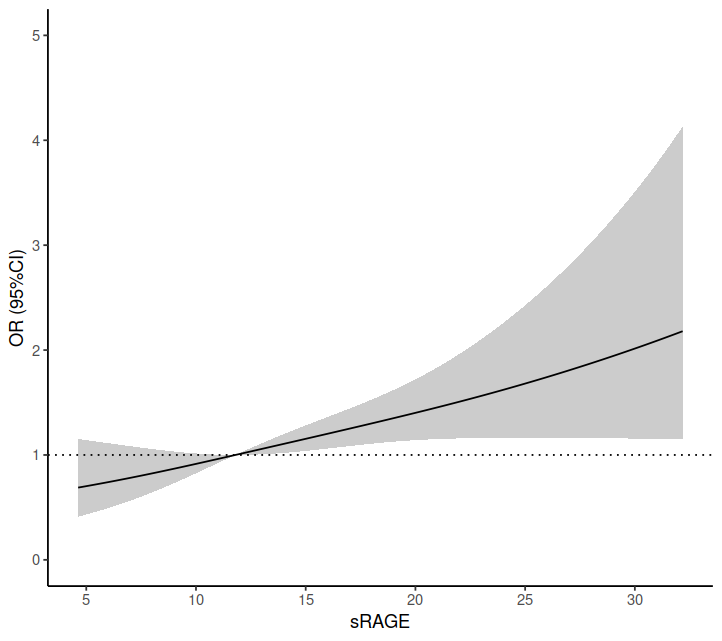

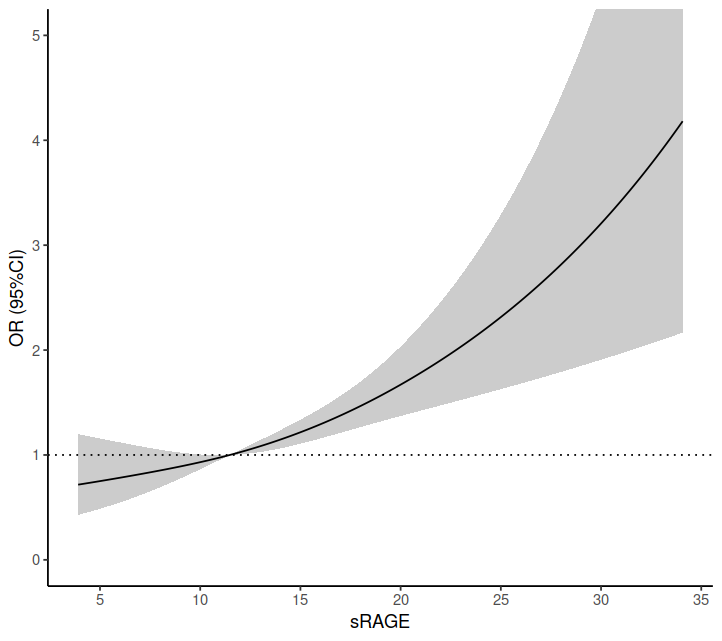


*non-linearity P=0.6*

*non-linearity P=0.8*

**B**

**A**

# **Fig. S2.** Association between soluble RAGE and incident coronary artery disease (**A**), stroke (**B**) and end-stage kidney disease (C) in individuals with type 1 diabetes and treatment-resistant hypertension. Unadjusted Cox regression models were fitted as a restricted cubic splines.


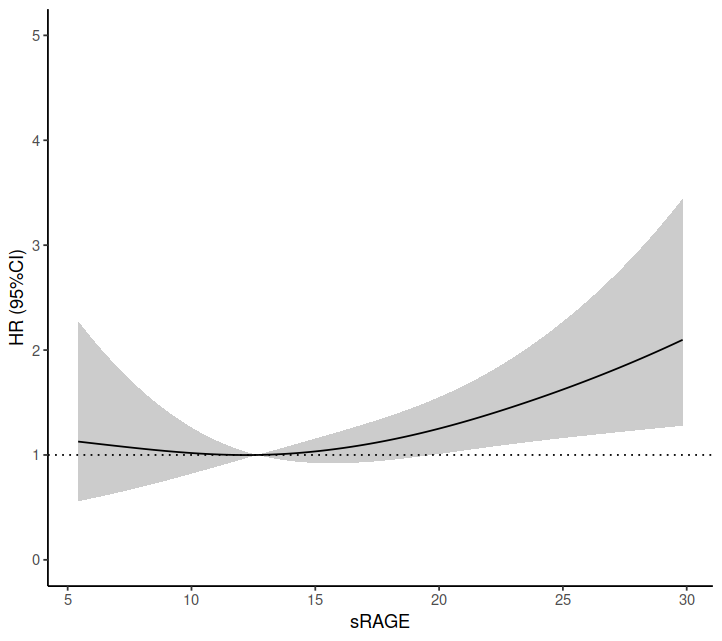

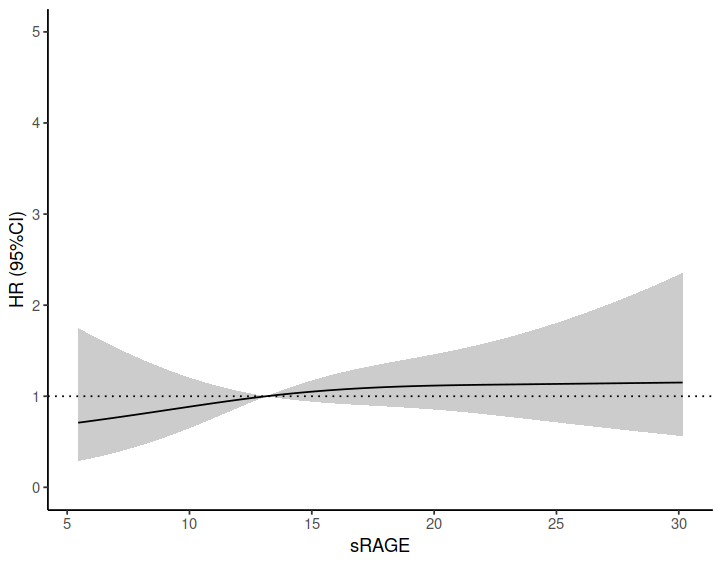


*non-linearity P=0.3*

*non-linearity P=0.6*

**A**

**B**


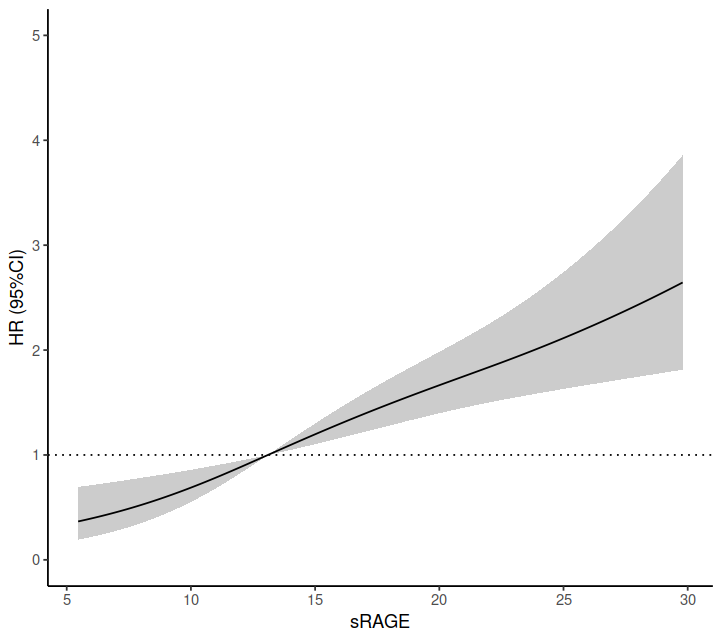


*non-linearity P=0.1*

**C**
